# Detection of Ablation Boundaries Using Different MR Sequences in a Swine Liver Model

**DOI:** 10.1007/s00270-022-03143-w

**Published:** 2022-04-21

**Authors:** Bennet Hensen, Urte Drenkmann, Bernd Frericks, Eva Rothgang, Marcel Gutberlet, Florian Länger, Wesley Gilson, Steffi Valdeig, Clifford R. Weiss, Frank Wacker

**Affiliations:** 1 Diagnostic and Interventional Radiology, Hannover Medical School, Hanover, Germany; 2 Diagnostic and Interventional Radiology, DRK Kliniken Westend, Berlin, Germany; 3 Industrial Engineering, Ostbayerische Technische Hochschule Amberg-Weiden, Weiden, Germany; 4 Department of Pathology, Hannover Medical School, Hanover, Germany; 5 Center for Applied Medical Imaging, Siemens Corporate Research, Baltimore, MD USA; 6 Division of Vascular and Interventional Radiology, Russell H. Morgan Department of Radiology and Radiological Science, Johns Hopkins University, Baltimore, MD USA; 7 The Johns Hopkins Center for Bioengineering Innovation and Design (CBID), Johns Hopkins University, Baltimore, MD USA; 8 STIMULATE-Solution Centre for Image Guided Local Therapies, Magdeburg, Germany

**Keywords:** MRI, MRI intervention, Interventional oncology, Thermal ablation, RF-ablation

## Abstract

**Purpose:**

To determine the magnetic resonance (MR) sequences best suited for the assessment of ablation zones after radiofrequency ablation (RFA).

**Methods:**

Three percutaneous MR-guided RFA of the liver were performed on three swine. Four pre-contrast and two hepatobiliary post-contrast sequences were obtained after ablation. Tissue samples were extracted and stained for nicotinamide adenine dinucleotide diaphorase hydride (NADH) and with hematoxylin and eosin. Post-ablation MR images and NADH slides were segmented to determine the total ablation zone, their Dice similarity coefficient (DSC), and the contrast-to-noise ratio (CNR) of the visible ablation boundary to normal liver tissue.

**Results:**

Two distinct layers were combined to determine the ablation zone: an inner layer of coagulation necrosis and an outer layer defined as the peripheral transition zone. Corresponding zones could be found in the MR images as well. Compared to histology, the total area of the MR ablation zone was significantly smaller on the pre-contrast T1 images (*p* < 0.01) and significantly larger with T2 turbo spin-echo (*p* = 0.025). No significant difference in size of the ablation zone depiction could be found between histology, post-contrast T1 volumetric interpolated breath-hold examination (VIBE), and post-contrast T1 3D Turboflash (TFL) as well as T2 SPACE images. All sequences but the pre-contrast T1 VIBE sequence showed a DSC above 80% and a high CNR.

**Conclusions:**

Post-contrast T1 3DTFL performs best when assessing ablation zones after RFA. Since the sequence requires a long acquisition time, T1 VIBE post-contrast offers the best compromise between acquisition time and estimation accuracy.

## Introduction

Image-guided thermal ablation has proven to be an effective, minimally invasive, local therapy [1, 2].

While effective, local recurrence of liver tumors after thermal ablation has been reported [3–5]. Therefore, the assessment of the exact extent of the ablation zone and the minimal ablative margin [6] using imaging is of utmost importance to ensure treatment of the entire tumor.

Studies have confirmed that ablation zones with clear margins of 5 mm beyond the tumor region provide less frequent recurrence [6, 7]. Post-ablation magnetic resonance imaging (MRI) can help provide both an accurate and precise assessment of the ablation zone.

Several soft-tissue contrasts, including T2-weighting and T1-weighting after administration of contrast agent, facilitate the assessment of the ablation zone [8–11]. However, previous studies evaluating the assessment of the ablation zones by MRI have either focused on single MRI sequences or used a single parameter such as total area, diameter, or distance in comparison to histopathology. Here, we choose 6 different MRI sequences which differ in tissue contrast, resolution, 3D/2D multislice acquisition, and acquisition time.

The purpose of this study was to evaluate and compare the performance of these different MRI sequences to assess the ablation margins after radiofrequency (RF)-ablation.

## Materials and Methods

### Animal Model

The Johns Hopkins University Animal Care and Use Committee approved this study (protocol number SW07M390). Three female domestic swine (mean body weight 45 kg) were anesthetized with an intramuscular injection of TKX (100 mg/ml telazol, 100 mg/ml ketamine and 100 mg/ml xylazine) at a dose of 1 mL per 22.8 kg body weight. The swine were intubated and mechanically ventilated with 1–2% isoflurane.

### MR-Guided Radiofrequency Ablation

All experiments were conducted on a 1.5 T MAGNETOM Espree MRI system using body and spine matrix coils, as well as the large four-channel flex coil (Siemens Healthineers, Erlangen, Germany). MR compatible RF-applicators (Celon ProSurge MR 150-T30, diameter 1.8 mm (15 G), electrode length 30 mm) were percutaneously inserted into the swine liver, using real-time imaging guidance with a balanced steady-state precession sequence (TR 547 ms, TE 2.5 ms, 510 Hz/Px, flip angle 70°, resolution 1.6 × 1.6 × 5 mm). RF-ablations were performed at 470 kHz with an active tip length of 30 mm, an input power of 30 W, and energy of 22 kJ at a mean duration of 20 min.

### MR Image Acquisition

Two ablations were performed in each swine for a total of 6 ablation zones. To assess the boundaries of the ablation zone, the following protocol was used after the final RF-ablation (see Table 1): First, a respiratory-triggered (liver scout) T2-weighted Sampling Perfection with Application optimized Contrasts using different flip angle Evolutions (SPACE) sequence and a respiratory-triggered T2-weighted 2D multi-slice Turbo Spin Echo (TSE) were performed.

**Table 1:** Imaging Protocol of the post-ablation imaging

Imaging sequence	T1 VIBE*	T1 3DTFL*	T2 SPACE	T2 TSE
TE (ms)	1.81	1.91	146	87
TR (ms)	5.27	1440	4300	4500
IR (ms)		260		
Flip angle (°)	9	15	150	150
Averages	0	3	2	1
FOV (mm)	320 × 240	337 × 450	260 × 260	280 × 280
Resolution (mm)	1.0 × 1.0 × 1.0	1.0 × 1.0 × 1.5	0.4 × 0.4 × 0.8	0.5 × 0.5 × 4.0
Matrix	320 × 240	337 × 450	640 × 640	512 × 512
Slice thickness (mm)	1.0	1.5	0.8	4.0
Number of slices	60	60	60	20
Bandwidth (Hz/pixel)	355	485	600	250
Triggering	Breathhold	Liver scout	Liver scout	Multibreathhold
*t*_Acq-av_ (min)	0.5	11	9	5

*TE* echo time, *TR* repetition time, *FOV* field of view, *Hz* hertz, *t*_Acq-av_ average acquisition time, *min* minutes

*Pre-and post-contrast imaging

For contrast enhancement, 0.1 ml/kg gadolinium ethoxybenzyl diethylenetriamine pentaacetic acid (Gd-EOB-DTPA, Primovist, Bayer-Schering Pharma, Berlin, Germany) was administered. For non-enhanced and post-contrast imaging, a respiratory-triggered T1-weighted 3D Turbo Flash (TFL) and a 3D T1-weighted Volumetric Interpolated Breath-hold Examination (VIBE) sequence were applied. The sequences were acquired in the same order for all animal experiments. Post-contrast T1-weighted VIBE and T1-weighted 3DTFL were acquired in hepatobiliary phase. The imaging plane of all MRI sequences was oriented perpendicular to the RF-applicator.

### Histopathology

The swine were euthanized under general anesthesia (~ 2–3 h after RFA) with a lethal intramuscular injection of sodium pentobarbital (Euthasol, Virbac). The liver was harvested. A wooden stick was inserted into the applicator path to help visualize the path and the ablation zone orientation inside the liver. Then, macroscopic tissue samples containing the thermal ablation zones were cut out en bloc perpendicular to the ablation zone. To prevent tissue shrinkage, the samples were immediately frozen in liquid nitrogen [12]. The frozen tissue samples were cut perpendicular to the RF-applicator into slices of 5 µm thickness. Two serial slices were processed and stained using hematoxylin and eosin (H&E) and Nicotinamide Adenine Dinucleotide Diaphorase Hydride (NADH), which is a marker of mitochondrial viability [8].

**Fig. 1:**
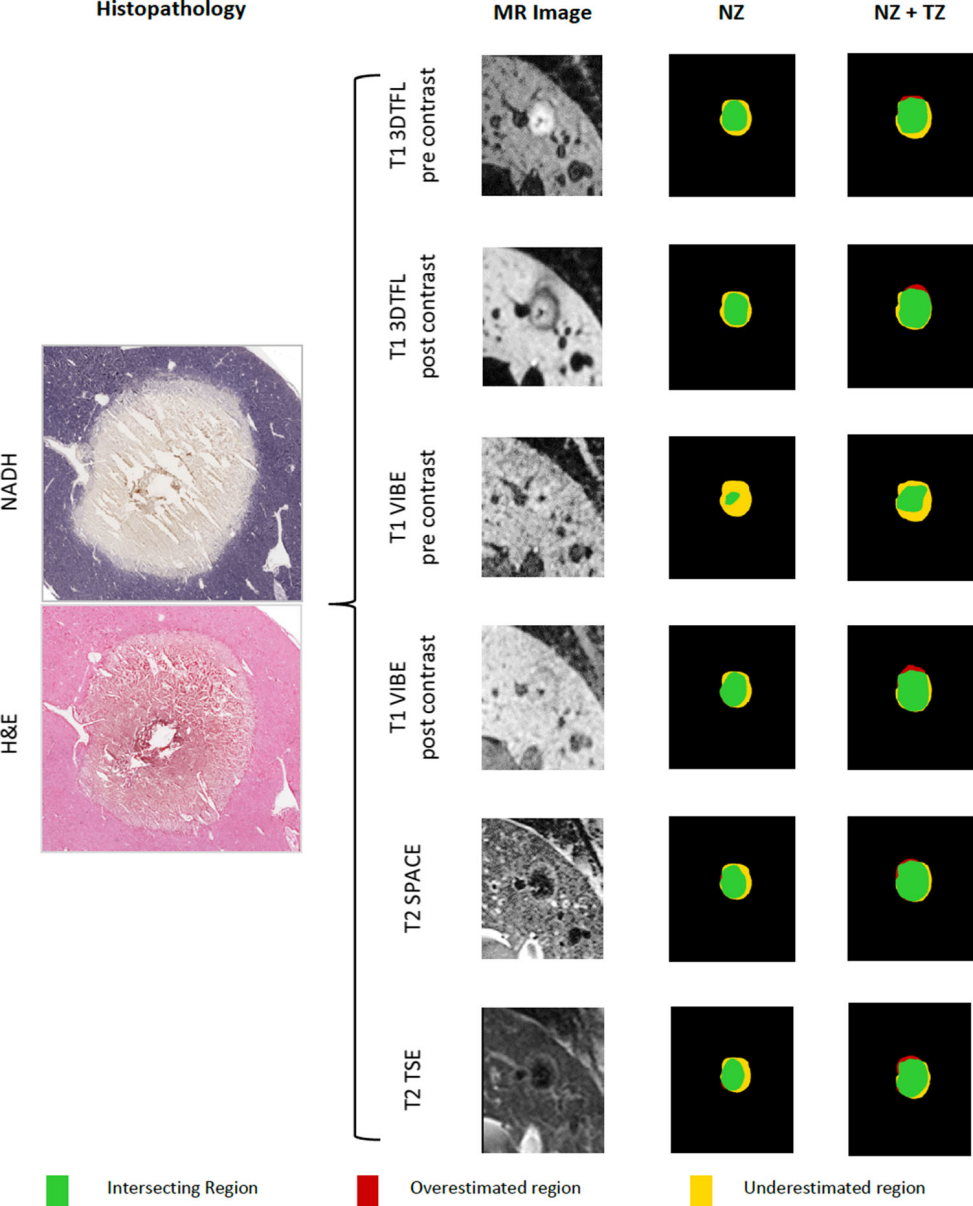
Histopathological images (NADH and H&E, left) and MRI post-ablation images (middle) for one RF-ablation of the liver in a domestic swine. Both, MRI post-ablation images and the corresponding NADH slide were segmented in their individual coagulation necrosis (NZ) and a corresponding peripheral transition zone (TZ). NZ and TZ together add up to the total ablation zone (TZ + NZ = AZ). The spatial overlap of each post-ablation image with the NADH slide for the NZ and the AZ area is displayed on the right side. The green area displays the intersecting region whereas the yellow area marks the region which was underestimated by the MRI post-ablation image. The red region displays the overestimation of the post-ablation MRI sequence

The samples were incubated at 37 °C for 45 min in a pH-adjusted buffer containing 0.8 mg/ml NADH and 1 mg/ml nitroblue tetrazolium (Sigma-Aldrich, St. Louis, MO). The samples were then washed with distilled water and further processed with 30%, 60%, 90% and 30% acetone washes before being rinsed with distilled water and covered with aqueous mount. Images of all samples were captured using a slide scanner (Aperio AT2, Leica Biosystems, Illinois) with 20-fold magnification (Fig. 1).

### Analysis of the Ablation Zone

**Fig. 2:**
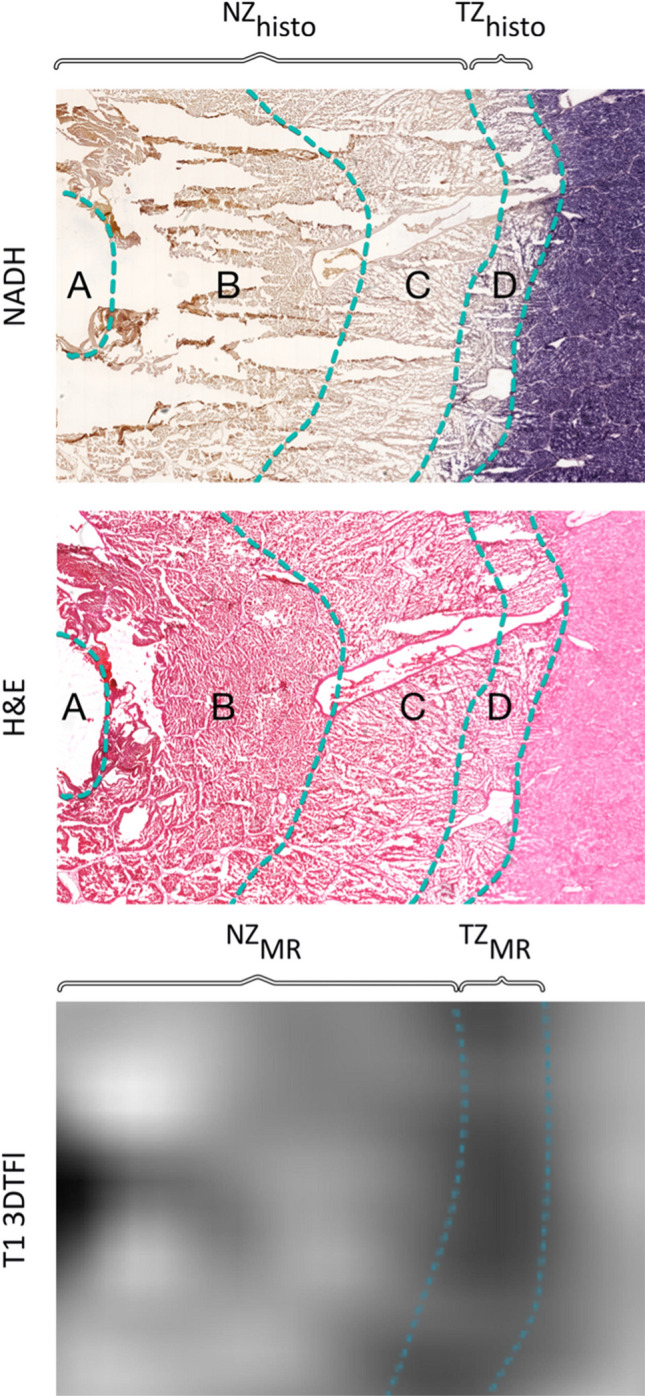
NADH and H&E images, showing the typical histopathological zones **A**–**D** and the zone segmentation depending on the NADH vitality staining. Zone A + B + C were combined as NZ_histo_ and zone D is the TZ_histo_. MR segmentation was conducted for each MRI sequence using the signal intensity. In this example, the hyperintense center was segmented as the NZ_MR_ in the corresponding MR image (T1 3DTFL post-contrast) and the hypointense peripheral zone adjacent to normal liver parenchyma was segmented as the TZ_MR_

Zone segmentation was based on the degree of hepatocyte staining [13, 14], which correlates with cell viability [15]. Similar to the procedure in Goldberg et al. [16], the central NADH negative “white zone” was segmented as the coagulation necrosis zone (zone A + B + C in Fig. 2 = NZ_histo_). This zone included the applicator path (zone A), a small per electrode coagulation necrosis (zone B), and a peripheral coagulation necrosis zone (zone C). Additionally, the light staining in the NADH slides between the NADH negative zone and normal liver parenchyma were segmented as the peripheral transition zone (zone D in Fig. 2 = TZ_histo,_).

Based on the image contrast of the six different MRI sequences, the coagulation necrosis zone (NZ_MR_) and a peripheral transition zone (TZ_MR_) were manually segmented by two experienced radiologists.

Using a custom-made Matlab R2018a (The MathWorks, Natick, United States) program, the histopathological and MR images were spatially aligned by visual inspection of the corresponding anatomical features (liver border, veins, arteries). The in-plane registration was performed by manually adapting the rotation, the spatial scaling, and translation of the overlaid MR image to the corresponding histological slice.

For further analysis, the NZ and the TZ provided by histology and MRI were combined in the ablation zone (AZ) [16].

### Spatial Overlap of the Ablation Zones

The spatial overlap of the ablation zone area (AZ_MR_) between the post-ablation MR images and the histopathology (AZ_histo_) was determined by the dice similarity coefficient (DSC) [17].To analyze whether MRI overestimated or underestimated the extent of the ablation zone, the ratio of the area of voxels identified as false positive and false negative in comparison to the ablation zone provided by histology was determined.

### Contrast-to-Noise Ratio

To measure the conspicuity of the TZ provided by post-ablation MRI, images were further analyzed regarding the underlying contrast-to-noise ratio (CNR) of the peripheral transition zone TZ to the surrounding liver tissue [18].

## Statistics

For statistical analyses, Matlab R2018a was used. The planimetric area of the individually identified ablation zones (AZ_MR_) and necrosis zones (NZ_MR_) of the six different post-ablation MR images was compared to the corresponding histopathology using a two-sided paired *t*-test. Assuming that a DSC ≥ 80% represents a good spatial overlap [19, 20], a one-sample right-sided *t*-test was used to determine whether the DSC for the different post-ablation images was significantly higher than that value. The CNR of the different post-ablation sequences was compared using a one-way ANOVA and a two-sample unpaired t-test. A significance level of 0.05 was chosen.

All calculated values are given as mean ± standard deviation (SD). The *p*-value is further visualized within the corresponding diagrams as asterisks: (*) *p* < 0.05, (**) *p* < 0.01 and (***) *p* < 0.001.

## Results

### Analysis of the Ablation Zone

In total, 10 histological NADH + H&E pairs as well as the corresponding post-ablation MR images were analyzed: 2 pairs from pig 1, 6 pairs from pig 2, 2 pairs from pig 3.

### Histopathology

The ablation zone was segmented into the coagulation necrosis NZ_histo_ and the peripheral transaction zone (TZ_histo_) depending on the NADH staining. The large central NADH negative zone (zone A + B + C) was segmented as the coagulation necrosis NZ_histo_.

The peripheral transition zone immediately surrounded the NZ_histo_ zone in all samples. This zone, named TZ_histo_, was characterized by light staining in the NADH slides and optically intact hepatocytes.

### MRI

Depending on the chosen sequence, the coagulation necrosis zone (NZ_MR_) was either hyperintense (T1VIBE and T1 3DTFL, pre, and post) or hypointense (T2 SPACE, T2 TSE). The transition zone (TZ_MR_) was hypointense on the pre-and hepatobiliary post-T1VIBE as well as T1 3DTFL, while it appeared hyperintense on T2 SPACE and T2 TSE.

### Comparison between MRI and Histopathology

Individual images of the post-contrast ablation sequences as well as the histopathology together with a colored overlay of the marked AZ and the peripheral TZ are shown in Fig. 1.

**Fig. 3:**
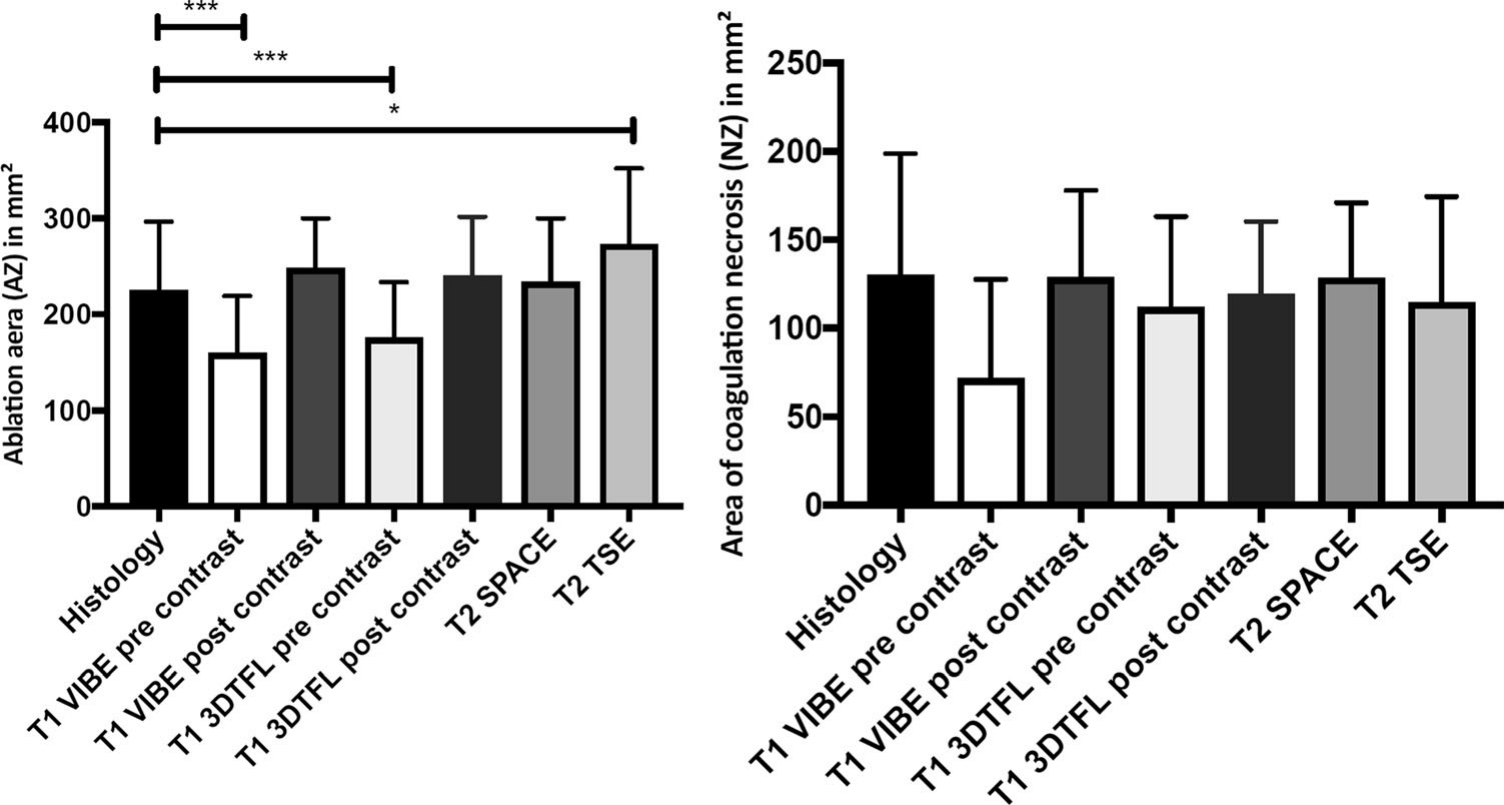
**A** Ablation zone area of the histological images (AZ_Histo_) compared to the post-ablation MR sequences in mm^2^ (*) *p* < 0.05, (**) *p* < 0.01. **B** Area of coagulation necrosis (NZ) of the histological images and the post-ablation MR sequences in mm^2^

The area of the AZ from histopathology and post-ablation MR images are summarized in Table 2 and Fig. 3. The mean ablation zone (AZ_histo_) area of the histological images was 226 ± 71mm^2^. T1 VIBE pre-contrast (*p* = 0.005) and T1 3DTFL pre-contrast (*p* = 0.002) showed a significantly different AZ area compared to the histological images. A two-sample right-sided paired *t*-test further revealed a significantly smaller ablation zone area for pre-contrast T1 VIBE (*p* = 0.0025) and pre-contrast T1 3DTFL (*p* = 0.001). The T2 TSE (*p* = 0.025) showed a significantly larger ablation zone area.

**Table 2:** Areas of the ablation zones of the histopathology and the post-ablation MR images

	Ablation zone area (NZ + TZ)	Coagulation necrosis zone area (NZ)
Histology	226mm^2^ ± 71mm^2^	130mm^2^ ± 69mm^2^
T1 VIBE pre-contrast	160mm^2^ ± 59mm^2^	72mm^2^ ± 56mm^2^
T1 VIBE post-contrast	248mm^2^ ± 52mm^2^	129mm^2^ ± 49mm^2^
T1 3DTFL pre-contrast	176mm^2^ ± 58mm^2^	112mm^2^ ± 51mm^2^
T1 3DTFL post-contrast	240mm^2^ ± 62mm^2^	119mm^2^ ± 41mm^2^
T2 SPACE	234mm^2^ ± 66mm^2^	129mm^2^ ± 42mm^2^
T2 TSE	274mm^2^ ± 79mm^2^	115mm^2^ ± 60mm^2^

The mean area of the NZ_histo_ was 130.01mm^2^ ± 69.85mm^2^ (see Fig. 3). No difference in the area of the NZ between the histopathology and the post-ablation MR images was found.

### Spatial Overlap of the Ablation Zone

**Fig. 4:**
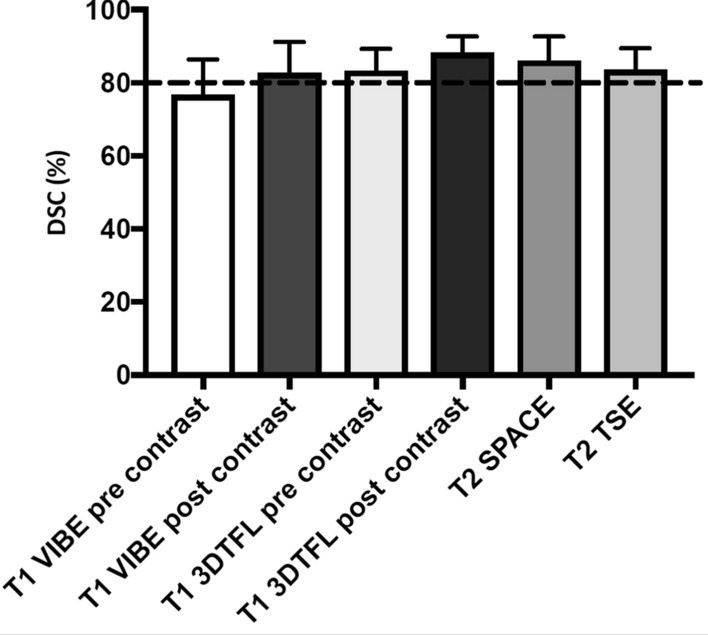
DSC value of the different sequences describing the respective spatial ablation zone (AZ_MR_) overlap with histopathology (AZ_histo_). The dotted line corresponds to a dice score of 80%, the threshold for an acceptable spatial overlap

The DSC values (see Fig. 4), describing the spatial overlap of the AZ area between the post-contrast ablation images and the histological images were not significantly higher than 80% for T2 TSE (83.6 ± 5.9%; *p* = 0.06), T1 VIBE pre-contrast (76.7 ± 9.7%; *p* = 0.15), T1 VIBE post-contrast (82.7 ± 8.5%; *p* = 0.17) and T1 3DTFL pre-contrast (83.4 ± 5.9%; *p* = 0.05), but were significantly higher for T2 SPACE (86.1 ± 6.6%; *p* < 0.01) and T1 3DTFL post-contrast (88.3 ± 4.5%; *p* < 0.001).

**Fig. 5:**
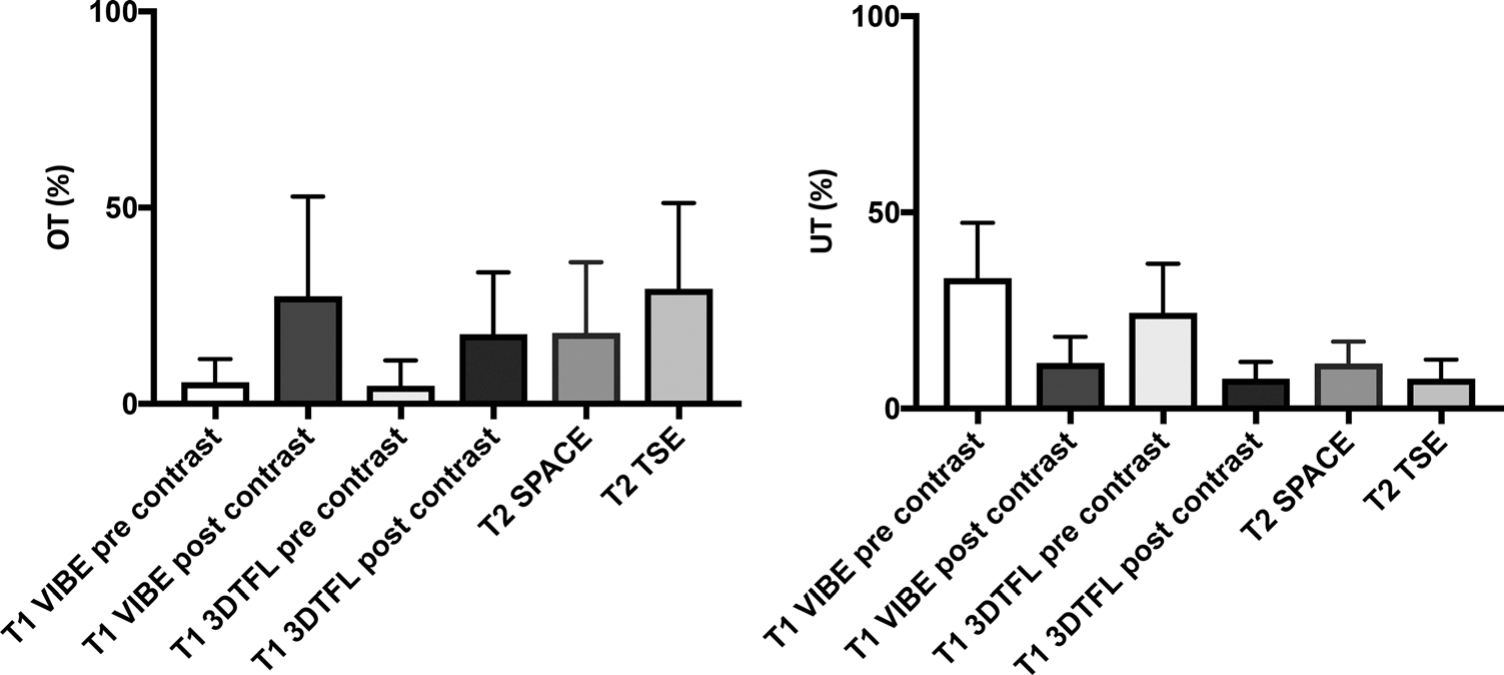
Overestimating (OT; left) and underestimating (UT; right) the area of the ablation zone (AZ_MR_) for the different post-contrast ablation images compared to the histopathology (AZ_Histo_)

Figure 5 shows the ratios of the overestimating and underestimating areas for the different post-contrast ablation images. OT ratios for the post-contrast images (OT T1 VIBE post-contrast: 27.4 ± 25.4%; OT T1 3DTFL post-contrast: 17.8 ± 15.8%) were higher than for the pre-contrast images (OT T1 VIBE pre-contrast: 5.5 ± 5.9%; OT T1 3DTFL pre-contrast: 4.6 ± 6.5%). The UT ratio, on the other hand, was generally higher for the pre-contrast images (UT T1 VIBE pre-contrast: 33.3 ± 14.1%; UT T1 3DTFL pre-contrast: 24.5 ± 12.6%). The T2 weighted sequences tended to overestimate the histological ablation zone (UT T2 TSE 7.6 ± 4,9%; OT TSE T2 30.1 ± 21.7%; UT T2 SPACE 11.5 ± 5.6%; OT T2 SPACE 18.1 ± 18.1%).

### Contrast-to-Noise Ratio

**Fig. 6:**
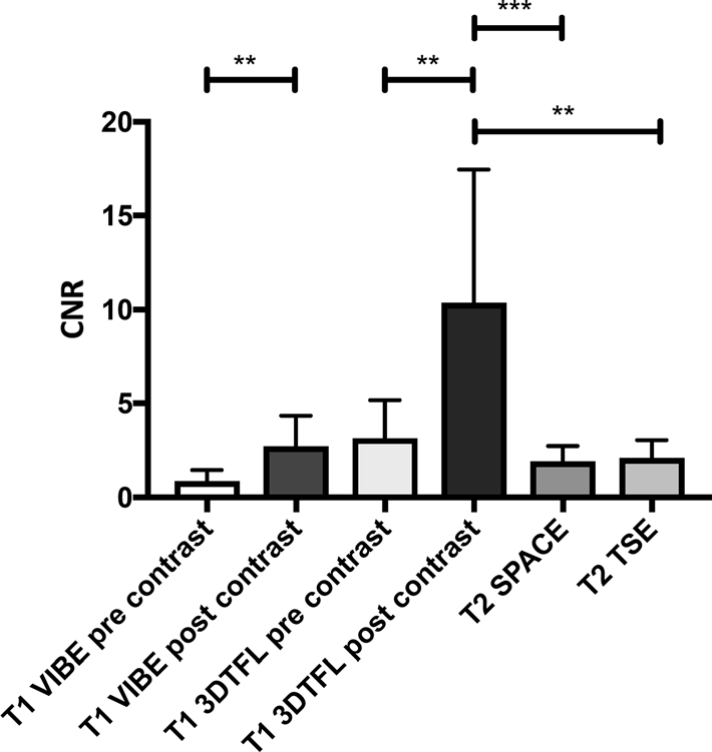
CNR of the TZ_MR_ in relation to the surrounding liver tissue for the different post-contrast ablation sequences. (*) *p* < 0.05, (**) *p* < 0.01 and (***) *p* < 0.001

One-way ANOVA revealed a significant difference for the CNR of the TZ_MR_ in relation to the surrounding liver tissue among the different post-contrast ablation MR images (*p* < 0.0001, Fig. 6). Comparing pre-and post-contrast images, the right-sided *t*-test revealed a significantly higher CNR for the post-contrast images of the T1 VIBE (CNR_PreContrast_ = 0.87 ± 0.57; CNR_PostContrast_ = 2.72 ± 1.63; *p* < 0.01) and T1 3DTFL (CNR_PreContrast_ = 3.15 ± 2.03; CNR_PostContrast_ = 10.38 ± 7.08; *p* < 0.01). The CNR of the T1 3DTFL post-contrast was significantly higher than the T2 SPACE (1.90 ± 0.82; *p* < 0.001) and the T2 TSE (2.09 ± 0.96; *p* < 0.01). No significant differences were found between the T1 VIBE post-contrast, the T2 SPACE, and the T2 TSE utilizing the one-way ANOVA (*p* = 0.31).

## Discussion

The accuracy of MRI sequences to determine the zone of tumor ablation is crucial as complete tumor removal yields lower local tumor recurrence rates [21]. Both T1 pre-contrast sequences underestimated the histologic ablation zone in contrast to the corresponding post-contrast sequence. Additionally, The T1 VIBE pre-contrast sequence had the lowest CNR/DSC and thus, should not be used to assess the ablation zone after RF-ablation. Nevertheless, it is possible to use the pre-contrast T1 VIBE sequence in clinical routine shortly after the ablation as a fast evaluation tool, as non-enhanced T1 sequences provide a high-resolution view of the tumor, RF applicator, and the surrounding anatomy. The ablation zone is also partly visible, which helps in the preliminary assessment of the therapy success [22, 23] without the need for administration of repeatable doses of contrast medium.

The varying areas of the ablation zone with different MRI sequences may be influenced by the detection sensitivity of the TZ_MR_ with MRI, which is influenced by the CNR. The highest CNR and DSC were measured for the hepatospecific T1 3DTFL post-contrast sequence. This could be due to the characteristic pattern of a markedly hypointense non-enhancing rim due to destroyed hepatocytes, which has also been observed in other studies [1, 24, 25]. However, the long acquisition of this high-resolution sequence is challenging in an interventional workflow. Therefore, the accurate T1 VIBE hepatobiliary post-contrast sequence could be a more feasible alternative in the clinical realm.

T2 weighted sequences are important for the assessment of residual hyperintense tumor tissue after ablation [24]. In this work, the T2 SPACE and T2 TSE sequences reached high DSC; however, both sequences tended to overestimate the histological ablation zone. These results are in agreement with the literature in liver rabbit models [24, 25].

The TZ is especially important for the clear demarcation of the ablation zone. It corresponds to the transition from NZ to viable hepatic tissue. The TZ_MR_ was hypointense on T1 weighted post-contrast images due to the dysfunctional necrotic tissue structure with dysfunctional hepatocytes, leading to impaired absorption of liver-specific Gd-EOB-DTPA and hyperintense on T2 weighted sequences. The hyperintensity of T2 weighted images is explained by water protons that are pushed to the periphery of the ablation zone [26], which other studies have described as changes in the bound water fraction [27]. Additionally, a transient hyperemia could be present as a hyperintense rim on T2 and T1 weighted post-contrast images [8]. Thus, the reason for the overestimation of the T2 weighted images could be due to peripheral edema and hyperemia, which constitutes a normal physiologic response to heating of tissue [16]. It is important to mention that for both T1 weighted post-contrast and T2 weighted sequences, these characteristics represent a challenge in determining complete ablation in the early phase since residual tumors can have the same MR imaging characteristics and could therefore be hidden in the TZ_MR_. In addition, TZ_MR_ may also contain tumor tissue that has been only transiently injured but could recover viability [28].

Despite the promising results of the T2 SPACE sequence, there are drawbacks for clinical use, as the acquisition time is very long. An alternative T2 weighted sequence that better fits the clinical workflow is the T2 TSE sequence. However, both sequences are very different regarding their T2 weighting, their resolution (2D multi-slice vs. 3D), and slice thickness. Using a 3D sequence, it is easier to assess the ablation zone in a multiplanar reconstruction, which is not possible using a non-isotropic 2D multi-slice sequence.

They are some limitations of the study: First, the small number of animals and ablations limit the statistical analysis. Second, the results obtained in healthy swine livers without a tumor model are different from those obtained in real patients with tumors surrounded by pathological liver tissue with cirrhosis, fibrosis, or steatosis. Third, registration between in-vivo MRI and histopathology is challenging. Anatomical landmarks were used to ensure both the validity of size and the correct spatial alignment between the MR imaging and pathologic images; however, precision is always hampered by the difficulty to obtain pathologic sections that correspond to the exact slice positioning of MR images. Other considerations might include partial volume artifact due to differences in slice thickness, which varied from 0.8 mm up to 4 mm. Fourth, we used only RF-ablation. The use of a single ablation device with unique settings deters from overall generalizability of the study findings and conclusions. Other ablation techniques like microwave ablation or high-intensity focused ultrasound may yield different results.

In conclusion, the study presented herein shows that 3D T1 weighted hepatobiliary post-contrast images and T2 weighted images are suited to assess the ablation zone after RF-ablation in a swine model due to their high DSC compared to the histology. Local tumor progression is more likely when post-ablation sequences overestimate the ablation zone. When post-ablation images underestimate the ablation zone, which is the case for the post-contrast T1 VIBE sequence and the T2 weighted sequences, the extent of actual treated area might be beyond expectation, thus minimizing risk for local tumor progression.

## Abbreviations

3DTFL: Three-dimensional turboflash (fast low angle shot)
ANOVA: Analysis of variance
bSSFP: Balanced steady state precession
CNR: Contrast-to-noise ratio
DSC: Dice similarity coefficient
H&E: Hematoxylin and eosin
MR(I): Magnetic resonance imaging
RF(A): Radiofrequency ablation
NADH: Nicotinamide adenine dinucleotide diaphorase hydride
NZ: Necrosis zone
SD: Standard deviation
VIBE: Volumetric interpolated breath-hold examination
SPACE: Sampling perfection with application-optimized contrasts using different flip angle evolution
TSE: Turbo spin echo (imaging)
TZ: Transition zone
